# The potential use of acylglycerols on the thermal inactivation of lactic acid bacteria for the manufacture of long-life fermented products

**DOI:** 10.1186/s12866-022-02694-9

**Published:** 2022-11-26

**Authors:** Luis Huerta-González, Fernando López-Valdez, Silvia Luna-Suárez

**Affiliations:** Food Biotechnology & Agricultural Biotechnology Labs. Centro de Investigación en Biotecnología Aplicada, Instituto Politécnico Nacional (CIBA-IPN). Tepetitla de Lardizábal, Tlaxcala, 90700 México

**Keywords:** Thermal death rate, Lactic acid bacteria, Acylglycerols, Fermented products

## Abstract

The effect of acylglycerols on the thermal inactivation of lactic acid bacteria used in the production of fermented products was studied. The starting point was the observation of an increase in thermal sensitivity in the presence of an emulsifier based on mono- and diacylglycerols in the culture medium. Analysis of the emulsifier showed that monoacylglycerols were the compounds responsible for this effect, with monopalmitin being the main contributor. Monostearin, on the other hand, showed significantly less potentiating effect. Interestingly, monoacylglycerols showed a greater bactericidal effect when used individually than when used in combination. On the other hand, the rate of thermal inactivation observed in reconstituted skim milk emulsions was lower than in peptone water emulsions, showing that the presence of proteins and colloidal particles increased the resistance of bacteria to heat treatment. With respect to pH values, a reduction in pH from 6.6 to 5.5 promoted an increase in the rate of thermal death. However, at pH = 5.5, the enhancing bactericidal effect was only detectable when the heat treatment was performed at low temperatures but not at high temperatures. This finding is of interest, since it will allow the design of moderate heat treatments, combining the use of temperature with the addition of acylglycerols, to prolong the shelf life of products fermented with lactic acid bacteria, and minimizing the destruction of desirable compounds that were obtained by the fermentation process.

## Introduction

The effect of lipids on the thermal inactivation of bacteria and spores has been widely reported in non-aqueous heating media [1, 2]. Under these conditions, lipids have been observed to increase the resistance of bacteria to heat, which has led to the generally accepted belief that lipids can protect bacterial cells and spores from possible damage caused by heat treatment. However, not all lipids appear to have the same protective effect. Such is the case of monoacylglycerols, which show bactericidal activity, so they have been studied for the inactivation of pathogenic organisms, either Gram-positive such as *S. aureus* and *L. monocytogenes*, or Gram-negative such as *S. typhimurium* [3–6]. However, very few studies have been conducted to determine the use of monoacylglycerols in conjunction with possible heat treatment. In addition, research has focused on the use of monolaurin (C12:0-MG), considered the monoacylglycerol with the highest bactericidal activity [7].

The application of monolaurin in food systems has focused on its incorporation into the final product, but its use throughout the manufacturing process has not been considered [8]. This is because, if monolaurin were to be used in a product whose process includes heat treatment, its hydrolysis could occur and consequently the production of undesirable flavours [9, 10]. Therefore, it is of interest to consider the use of other monoacylglycerols in addition to monolaurin, but little information is available on these compounds.

The use of monoacylglycerols with apparently lower bactericidal activity than monolaurin could have certain advantages, for example, in the manufacture of long-life fermented products. In these products, a post-fermentation heat treatment is used to prolong the shelf life by inactivating lactic acid bacteria, their enzymes, and other possible microorganisms such as yeasts and moulds. However, conventional heat treatment causes losses of nutrients and colours, in addition to altering flavour and texture properties [11, 12]. In this context, the combination of monoacylglycerols together with heat treatment could lead to less severe, but more efficient thermal processes, obtaining higher quality products. For example, heat treatments could be optimized to preserve the activity of the enzyme α-D-galactosidase, whose presence in fermented dairy products is particularly desirable for consumers deficient in that enzyme [13, 14].

## Materials and methods

### Strains and materials

Standard strains of *Streptococcus thermophilus*, *L. lactis subsp. lactis var. diacetylactis*, *Lacticaseibacillus rhamnosus* [15] and *Lactobacillus delbrueckii subsp. bulgaricus* were obtained from Unipath Ltd. All culture media were obtained from Unipath Ltd. Lactose was obtained from Difcon Laboratories.

### Preparation of emulsions

Reconstituted skim milk emulsions (RSM) were prepared according to Table 1. Corn oil (MO), palm oil (PO), anhydrous milk fat (AMF) or hydrogenated palm kernel oil (HPKO) were incorporated as fat source. RECODAN™ (Danisco A/S, Denmark) was used as the emulsifying agent (C.E.). Emulsification of the ingredients was performed at 70—75 °C using a Rannie single-stage homogenizer at a homogenizing pressure of 100 bar [16]. Fat particle size was standardized to a mode between 1.23 μm and 1.58 μm in all prepared emulsions using a Malvern particle and droplet size sizer, 2600c series (Malvern Instruments, Malvern). All emulsions were sterilized at 121 °C for 15 min. Emulsions containing pure mono- and diacylglycerols, instead of the emulsifying agent, were also prepared using a Silverson model L2R high-shear homogenizer instead of the Rannie single-stage homogenizer. To identify the effect of the presence or absence of proteins in the medium, emulsions were prepared with peptone water (PW) according to Table 1, to evaluate the heat treatments and compare them with those developed in RSM emulsions.

**Table 1:** Composition of different emulsions for thermal treatment trials (% weight)

	Skim milk	Emulsifier	Fat	Peptone	Lactose	Pure
Emulsion	powder	(C.E.)	(4 types)	Water		Water
Reconstituted skim milk (RSM)	8.5					91.5
RSM + C.E	8.5	0.2				91.3
RSM + C.E. + fat	8.5	0.2	4			87.3
RSM + fat	8.5		4			87.5
Peptone water (PW)				100		
PW + C.E		0.2		99.8		
PW + lactose				95.9	4.1	
PW + C.E. + lactose		0.2		95.7	4.1	
PW + C.E. + lactose + fat		0.2	4, 15, 30	91.7, 80.7, 65.7	4.1	

### Inoculation of emulsions

Microbial suspensions of each bacterium containing 10^9^ to 10^10^ CFU mL^−1^ were prepared by the addition of a DVI-type starter culture to 10 mL of reconstituted skim milk at 25 °C [17]. The suspension was allowed to reactivate for 1 h at 37 °C. After that time, 1 mL of the microbial suspension was taken and added to 99 mL of each emulsion previously prepared and sterilized according to Table 1, now considered as heating medium.

### Determination of thermal death parameters of lactic acid bacteria (LAB)

Decimal reduction times (D-values) and the number of degrees the temperature must be increased to achieve a tenfold reduction in D-value (Z-values) were determined according to Reveron et al. [18] and Shearer et al*.* [19].

Heat treatments were performed at 62.5 °C for all strains, using RSM and RSM + C.E. as the heating medium. Then the lactic acid bacterium showing the highest thermal resistance (*S. thermophilus*) was used to perform subsequent heat treatments at 60 °C, 65 °C and 68 °C varying the source of fat (Corn oil (MO), palm oil (PO), anhydrous milk fat (AMF) or hydrogenated palm kernel oil (HPKO). Two different strains of *S. thermophilus* were used, codes TA054 and TA060, in order to know if the bacterial strain had an influence on the D values during heat treatments in RSM emulsions,

TA060 *S. thermophilus* strain was used to study the effect of several concentrations of monomyristin (C14:0), monopalmitin (C16:0) and monostearin (C18:0), as it showed the highest thermal resistance. The presence of protein and the effect of pH on the synergistic bactericidal effect of monoacylglycerols with heat treatments were assessed varying the heating media composition according to Table 1. All tests were performed in quadruplicate and both the mean and standard deviation were calculated. ANOVA test was carried out at *p* < 0.05.

### Enumeration of survivors

Number of survivors to heat treatment was determined by surface plating using the spiral plating technique [20]. For *S. thermophilus*, plating was performed on M17 medium, followed by aerobic incubation at 37 °C for 48 h. For *L. lactis subsp. lactis var. diacetylactis*, plating was also performed in M17 medium, but followed by aerobic incubation at 30 °C for 48 h. On the other hand, acidified MRS medium was used for *L. delbrueckii subsp. bulgaricus*, followed by anaerobic incubation at 37 °C for 72 h, and finally, *L. rhamnosus* used acidified MRS medium followed by incubation at 45 °C for 72 h. Colony counting was performed using an automatic laser colony counter (Spiral System Instruments Inc.).

## Results and discussion

### Thermal death parameters of LAB species in RSM emulsions

**Fig. 1:**
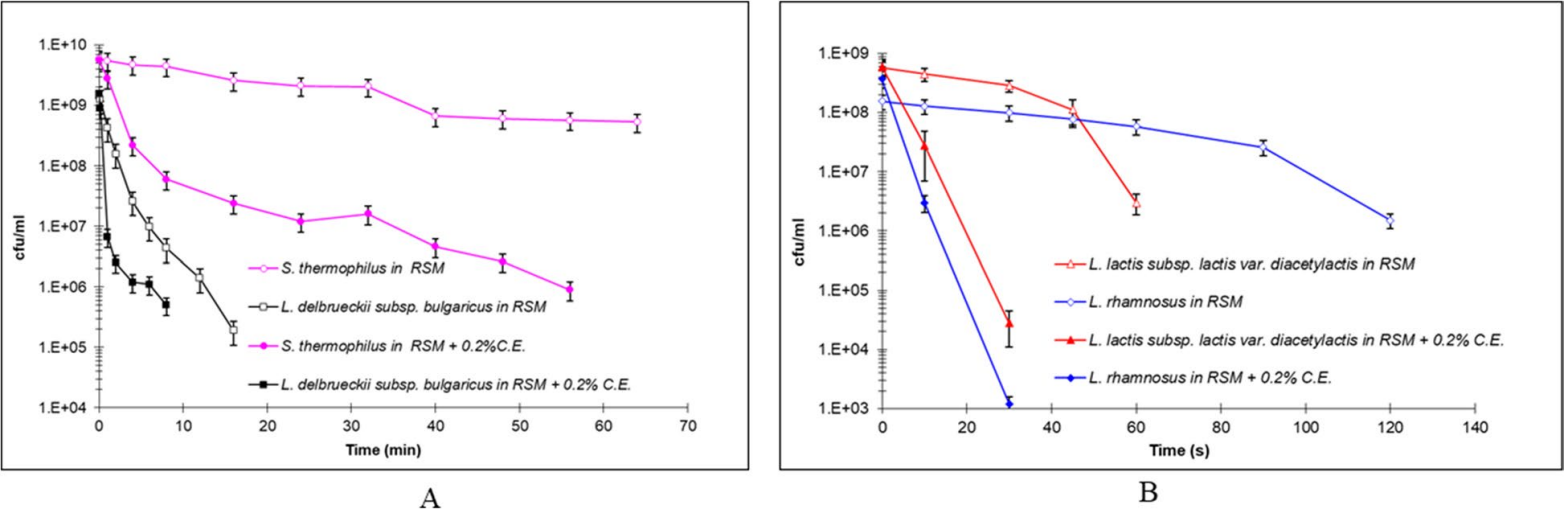
Thermal death curves at 62.5 °C of LAB species in RSM emulsions and RMS + 0.2% C.E. emulsions. **A** *S. Thermophilus* and *L. delbrueckii subsp. bulgaricus*; **B** *L. lactis subsp. lactis var. diacetylactis*, and *L. rhamnosus* (mean of quadruplicate essays)

Number of survivors during heat treatment at 62.5 °C, in RSM emulsions and RSM emulsions + 0.2% E.C., for *S. thermophilus*, *L. lactis subsp. lactis var. diacetylactis, L. rhamnosus* and *L. delbrueckii subsp. bulgaricus*, are shown in Fig. 1. Decimal reduction times (D values) are shown in Table 2. *S. thermophilus* presented the highest thermal resistance and allowed a better observation of the changes due to the presence of E.C., so further tests were focused on this species.

**Table 2:** D values (decimal reduction times) in seconds at 62.5 °C for the four species of LAB in RSM and RSM + 0.2% C.E. Average of 4 determinations. RSM: Reconstituted milk emulsions, C.E.: Commercial emulsifier

LAB species	RSM	Std. Dev	RSM + 0.2% C.E	Std. Dev
*S. thermophilus*	3546	± 60.89	1104	± 77.4
*L. lactis* subsp. *lactis* var. *diacetylactis*	31	± 4.2	7	± 0.2
*L. rhamnosus*	68	± 1.1	6	± -
*L. delbrueckii* subsp. *bulgaricus*	138	± 8.4	84	± 4.2

### Thermal death parameters of S. thermophilus at different temperatures

D and Z values for *S. thermophilus* thermal death curves at 62.5 °C, 65 °C, and 68 °C for various emulsions are shown in Table 3. It was found that thermal resistance of bacterial cells decreased in the absence of fat and in the presence of E.C. For emulsions prepared with some type of fat, bactericidal effect of the emulsifier decreased, regardless of the type of fat used. Furthermore, the addition of emulsifier also modified the shape of the thermal death curve, resulting in a combination of two straight lines represented by I (the linear section closest to time zero) and II, respectively. Analysis of variance showed that D values obtained for RSM emulsions were the highest; there was no significant difference between the D and Z values obtained for RSM + 0.02% E.C. + any fat emulsions; and there was a significant difference in the D values, but not in the Z values for RSM + 0.02% E.C. emulsions.

**Table 3:** D values at different temperatures for *S. thermophilus* in RSM emulsions with different lipid composition, pH 6.6. RSM (mean of quadruplicate essays)

Temp (°C)	Linear	RSM	RSM + CE	RSM + CE	RSM + CE	RSM + CE	RSM + CE
	region			+ PO	+ MO	+ AMF	HPKO
60	I	327.6	111.7	300.2	343.1	312.8	329.4
62.5	I	59.1	4	47.5	47.6	49.2	47.8
	II		28.6				
65	I	11.1	1.8	8.7	8.1	8.4	9
	II		6.5				
68	I	1.4	0.4	0.9	0.7	0.8	0.8
Z values		3.4	3.4	3.2	2.9	3.1	3.2

### Influence of the bacterial strain used for the thermal experiments

Table 4 shows the D values obtained during heat treatment at 62.5 °C and 65 °C in RSM emulsions for two different strains of *S. thermophilus*, codes TA054 and TA060. Analysis of variance showed that an increase in 2.5 °C resulted in a significant reduction in D values for both strains. None significant difference in D values was obtained for the same bacterial strain in different emulsions. However, strain TA060 presented a higher thermal resistance than strain TA054.

**Table 4:** D values for *S. thermophilus* strains TA054 and TA060 (mean of quadruplicate essays; s.d. = standard deviation)

Strain	Temp (°C)	SMP + CE + PO	s.d	SMP + CE + MO	s.d	SMP + CE + AMF	s.d	SMP + CE + HPKO	s.d
TA054	62.5	11.1	± 0.46	12.2	± 0.93	11.5	± 0.29	11.2	± 0.36
TA054	65	3.8	± 0.49	3.1	± 0.27	3.2	± 0.14	3.4	± 0.10
TA060	62.5	47.5	± 1.90	47.6	± 2.22	49.2	± 1.13	47.8	± 1.01
TA060	65	8.7	± 0.15	8.1	± 0.30	8.4	± 0.20	9.0	± 0.21

The results highlighted the fact that the D values are specific to the bacterial strain used. Therefore, strain TA060 was used for the rest of the experiments.

### Emulsions containing different monoacylglycerol concentration

**Fig. 2:**
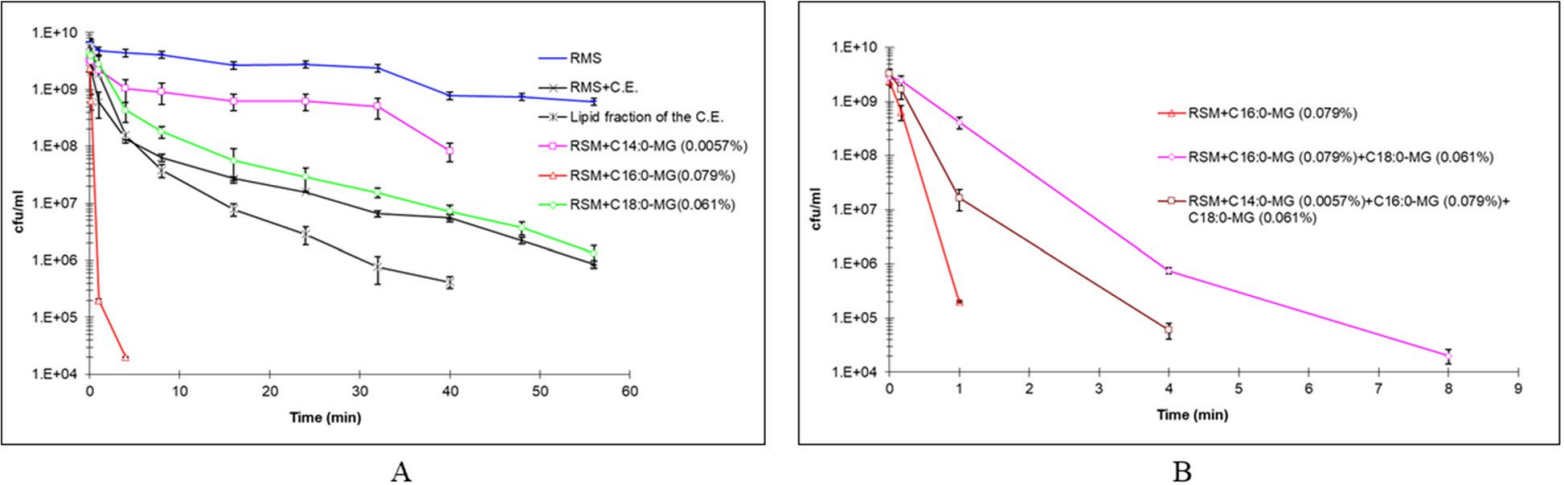
**A** Thermal death curves at 62.5 °C for *S. thermophilus* in RSM emulsions containing pure monoacylglycerols in the same concentrations as in the commercial emulsifier. pH 6.6. **B** Combined effect of onoacylglycerols in the thermal inactivation of *S. thermophilus*. (Mean of quadruplicate essays)

Figure 2A shows the thermal death curves of *S. thermophilus* at 62.5 °C in RSM emulsions containing monomyristin (C14:0), monopalmitin (C16:0) and monostearin (C18:0) at the same concentration as found in C.E. All monoacylglycerol fractions affected the bacterial thermal resistance to some extent. For the concentration of monomyristin present in C.E. (0.0057%), only a slight bactericidal effect additional to that of temperature could be observed. Monopalmitin showed the highest contribution to thermal inactivation, demonstrating that at a concentration as low as 0.079% in RSM emulsions, it was able to reduce the D value at 62.5 °C from 59.1 min to 0.3 min. Monostearin also showed a bactericidal effect in combination with temperature, although it was significantly lower than that achieved by monopalmitin.

**Fig. 3:**
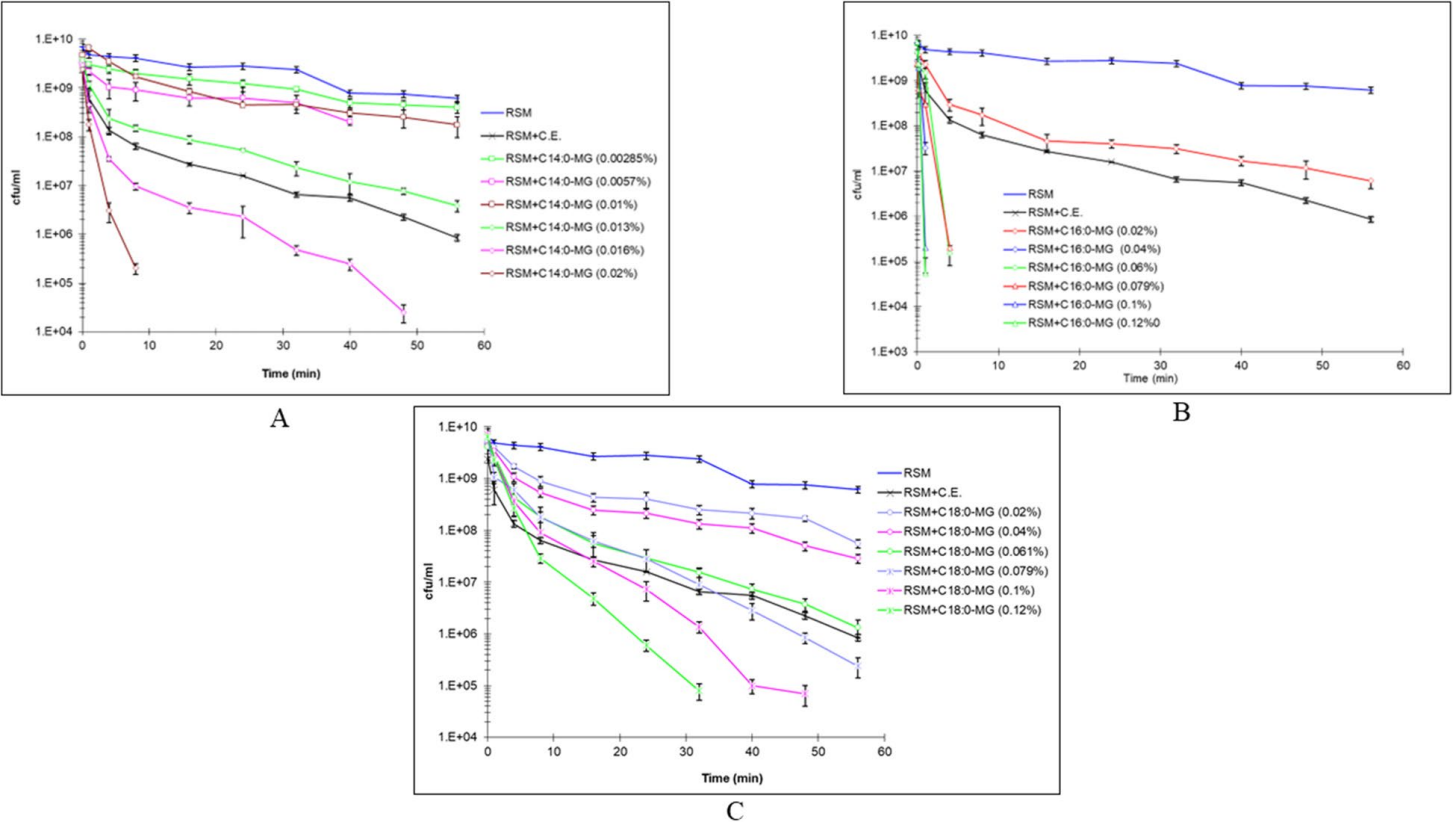
Thermal death curves at 62.5 °C for *S. thermophilus* in RMS emulsions containing different concentration of monoacylglycerols **A** monomyristin; **B** monopalmitin, **C** monoestearin. pH 6.6. (Mean of quadruplicate essays)

The combined effect of monoacylglycerols on the thermal inactivation of *S. thermophilus* is shown in Fig. 2B. The combination of monoacylglycerols did not increase the lethality of the process. On the contrary, the bactericidal effect was greater for pure monopalmitin than for the mixtures, indicating a possible competition in their mode of action. Thermal death curves for different concentrations of monomyristin, monopalmitin and monostearin are shown in Fig. 3. The amount of each monoacylglycerol required to promote a significant change in the thermal resistance of the culture was much lower for monomyristin than for monopalmitin or monostearin. The lethality curves indicated that a concentration as low as 0.013% monomyristin could increase the lethality of heat treatment by a factor of 2.

For monopalmitin, a concentration higher than 0.02% was necessary to achieve a similar effect. In the case of monostearin, a smaller increase in heat lethality was observed at a concentration of 0.02%. On the other hand, analysis of variance showed that the presence of monomyristin increased heat lethality significantly at concentrations below 0.02%. Higher concentrations increased the bactericidal effect to the point where no survivors could be detected at any time. In the case of monopalmitin, concentrations below 0.04% increased lethality progressively, and maximum lethality was achieved at a concentration of 0.04%. At higher concentrations, no further changes were observed. Finally, monostearin showed a much smaller effect on the heat lethality. Concentrations below 0.04% increased lethality faster than concentrations above this value.

Results confirmed that the shorter the monoacylglycerol chain, the better the bactericidal effect in combination with heat treatment. Undoubtedly, the bactericidal effect originally observed by the presence of C.E. and attributed to monopalmitin, was due to the higher amount of this acylglycerol in C.E.

### Effect of the presence of protein on the bactericidal action of monoacylglycerols

**Fig. 4:**
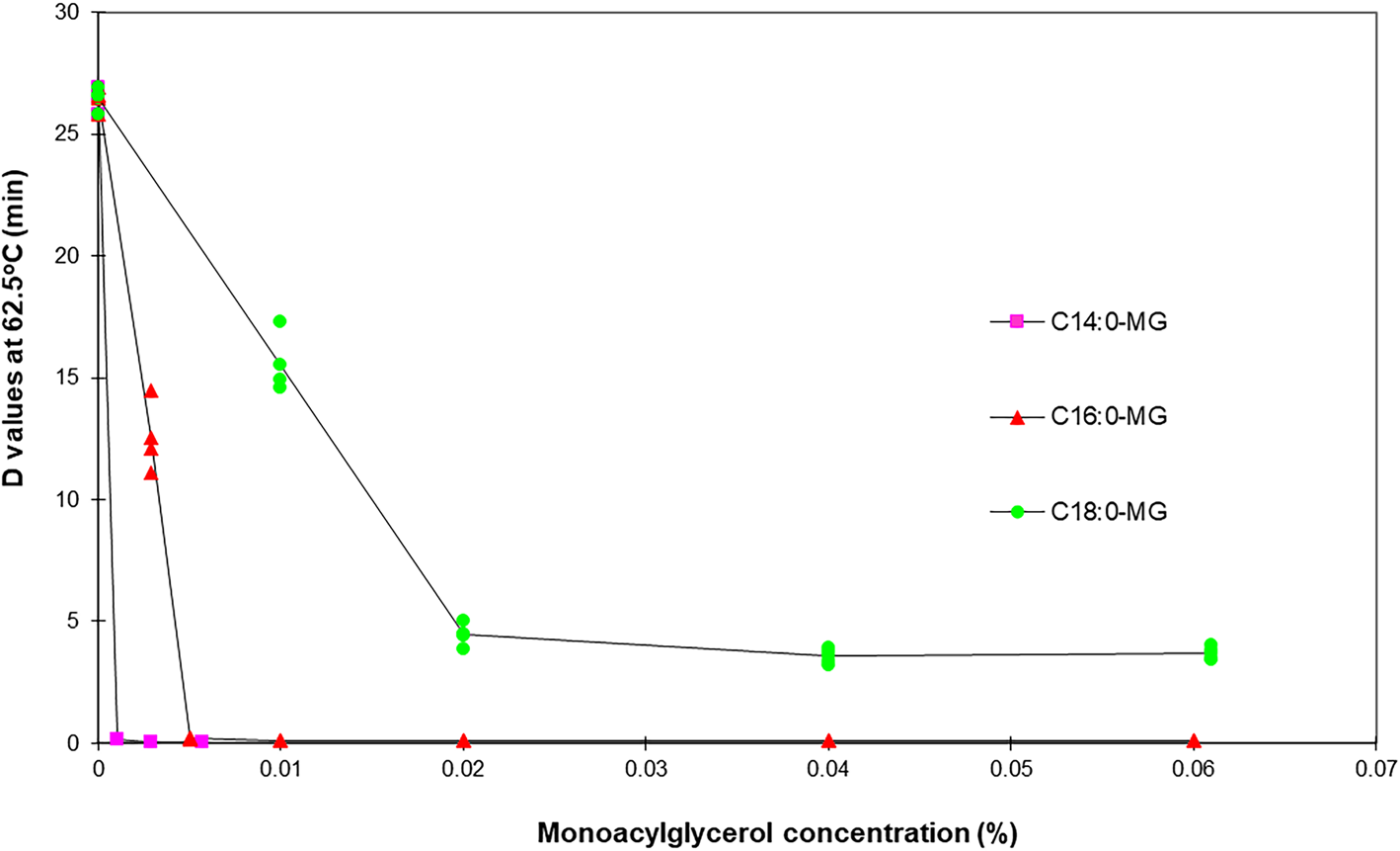
Relationship between D values al 62.5 °C for *S. thermophilus* at different monoacylglycerols concentrations in PW emulsions, pH 6.6. (Mean of quadruplicate essays)

To determine whether the bactericidal effect of monoacylglycerols in combination with heat treatment was affected by the presence of proteins, thermal death curves of *S. thermophilus* in peptone water emulsions (PW) were obtained. The relationship between D values at 62.5 °C and monoacylglycerol concentrations in PW emulsions is shown in Fig. 4. It was observed that the required concentration of monoacylglycerols to produce a bactericidal synergistic effect with heat treatment was much lower in PW emulsions than in RSM emulsions. Trace amounts of monomyristin (i.e., 0.001%) reduced the D value from 19.8 min to almost zero. For monopalmitin, the maximum effect was reached at a concentration of 0.005%. Concentrations above this level did not increase the lethality of the heat treatment significantly. With respect to monostearin, the maximum bactericidal effect was reached at a concentration of 0.02% and any subsequent addition of this monoacylglycerol did not increase the lethality of the heat treatment. These results confirmed the lower synergistic bactericidal effect of monostearin with respect to the other monoacylglycerols.

According with these observations, the concentration of monoacylglycerols required to increase the heat treatment lethality was higher in RSM emulsions than in PW emulsions. These differences are attributed to the presence of milk proteins, which probably reduced the interactions of monoacylglycerols with bacterial cells [21]. These findings contrast with those reported for monolaurin by Zhang et al. [22], who found that the antibacterial activity of monolaurin remained unchanged in the presence of protein.

### Effect of pH on the synergistic effect of monoacylglycerols with temperature

Determination of thermal death parameters in PW emulsions was extremely useful because pH values could be adjusted to a level that was not possible to achieve in RSM emulsions due to casein precipitation. Thus, thermal death curves of *S. thermophilus* in PW emulsions at pH 6.6 and 5.5 were obtained for two different temperatures.

The relationship between D values, pH and temperature is shown in Table 5. Analysis of variance showed that lower D values were achieved at lower pH values. In addition, a significant difference in D values for each temperature was observed.

**Table 5:** D values at different pH values for *S. thermophilus* in PW emulsions with different lipid composition, temperatures 60 °C and 65 °C (mean of quadruplicate essays)

pH	Temp (°C)	PW	PW + CE	PW + CE + PO	PW + CE + MO	PW + CE + AMF	PW + CE + HPKO
	60	75.8	26.6	60.4	46.9	62.4	58.0
6.6	62.5	23.8	4.8	11.5	8.8	11.2	11.8
	65	4.1	1.5	2.6	2.3	2.8	2.9
	68	0.5	0.3	0.4	0.4	0.4	0.4
Z value		3.6	4.2	3.7	3.9	3.7	3.7
R^2^		0.999	0.996	1.000	0.999	0.999	0.999
	60	37.1	15.8	21.7	17.3	25.6	26.2
5.5	62.5	10.4	4.7	5.3	5.4	6.0	6.9
	65	1.6	1.6	1.7	1.7	1.7	1.8
	68	0.7	0.4	0.4	0.5	0.5	0.5
Z value		4.8	5.1	4.9	5.3	5.1	4.7
R^2^		0.926	1.000	0.999	0.998	1.000	1.000

Above results showed the synergistic effect of monoacylglycerols (monopalmitin, monostearin, and monomyristin) in combination with heat treatment. On the other hand, once the exact E.C. composition was known, it was corroborated that there were no changes in the heat lethality as a result of the presence of diacylglycerols or fatty acids.

Addition of anhydrous milk fat or any vegetable fat reduced the bactericidal effect of monoacylglycerols. At concentrations of 15% and 30% fat content, bacterial heat resistance increased significantly, which agrees with the protective effect of fats observed by other authors [1]. All studied fats had the same protective effect in RSM emulsions.

Observations in RSM emulsions indicated that the synergistic bactericidal effect was greater for pure monoacylglycerols than for mixtures, indicating a possible competition in their mechanism of action. However, the monomyristin-monopalmitin-monostearin mixture did show a significant inhibitory effect in combination with heat treatment in RSM emulsions at concentrations as low as 0.01% monomyristin, 0.02% monopalmitin and 0.04% monostearin. These findings agree with those reported by Zhang et al. [7] for the combination of monomyristin and monolaurin, which caused cell lysis at high doses as a result of their combined action. Garcia et al. [3] also reported the bactericidal action of monocaprylin in combination with acetic acid, for the inactivation of *Listeria monocytogenes*.

From these results, it could be argued that the enhancing effect on the thermal inactivation of bacteria was not caused by a heat-enhanced heat injury of bacterial cells, but by a heat-enhanced chemical inactivation. That is, chemical inactivation by potentiation of the interaction of monoacylglycerols with the bacterial cell wall and cell membrane seems to be the most likely mechanism. This seems to agree with that reported by Yoon et al. [4], who pointed out that the lytic behavior of fatty acids and monoacylglycerols on the membrane derived from their amphipathic properties, which can lead to membrane destabilization and pore formation, with consequent inhibition of bacterial cell growth (bacteriostatic action) or cell death (bactericidal action).

*S. thermpilus* cell wall is mainly composed by C14:0 (1.62%), C16:0 (33.68%), C16:1 (3.53%), C18:0 (12.33%), C18:1–9 (5.72%), C18:1–11 (24.83%), ΔC19:0 (1.34%), C20:0 (1.8%), C20:1 (15.13%) [23]. These researchers reported that the presence of oleic acid in the growth medium caused an increase of this fatty acid in the cell membrane. It can be inferred that this lactic acid bacterium uses the available fatty acids to incorporate them into the membrane. Furthermore, Min et al. [24] demonstrated that *S. thermophylus* changed the cell membrane fatty acid composition to adapt it and survive to heat treatment. Taking into account these findings, together with the results obtained in the present work, it could be argued that the bacterial effort to incorporate monoacylglycerols instead of free fatty acids into the cell membrane could lead to a defective structure, resulting in an increased microbial sensitivity with the consequent death derived from the thermal treatment. Regarding C14 greater effect compared with C16 and C18, this could be attributed to the shorter chain length, showing a higher reactivity as a consequence of an increased polarization derived from the proximity between the acid protons and the hydrophobic chain.

In addition, fatty acids have the potential to disrupt the electron transport chain by binding to electron transporters or altering membrane integrity, as well as interfering with oxidative phosphorylation by decreasing the membrane potential and proton gradient [25].

Another possible mechanism is that the fatty acids that are part of monoacylglycerols can directly inhibit enzymes present in the membrane such as glucosyltransferase, presumably because their molecular structure is similar to that of fatty acids, so they could also associate with other proteins that are part of the membrane [26]. Finally, an additional mechanism derives from the fact that monoacylglycerols are surfactant compounds. In this regard, Furukawa et al. [27] reported that the addition of a surfactant to bacterial suspensions prevents the formation of microbial clumps formed during heat treatment and increases the inactivation rate.

Regarding the potential application of these results in the food industry, it is worthy to mention that heat treatments are widely used to increase the shelf life of food products since spoilage microorganisms are temperature sensitive and can be destroyed by the application of heat [28]. Technological advances in the design and operation of heat exchangers allow thermal processes to be controlled quite precisely with minimal energy loss [29]. However, most of the nutrients present in foods are affected by the application of heat, so it is common for nutritional deterioration to occur in thermally treated products. In the case of fermented dairy products, heat treatment aims to eliminate to a greater or lesser extent lactic acid bacteria, as well as yeasts and molds that are present as contaminants, avoiding post-acidification and proteolysis as a result of the metabolism of microorganisms during the storage period in their shelf life [30].

Heat resistance of lactic acid bacteria is highly dependent on the pH value of the medium. For example, it was observed that in yogurt at pH 4.55, 97.6% of thermophilic lactic acid bacteria were able to survive heat treatment at 65 °C for 22 s, whereas at pH 3.82, 99.99% of the same bacteria were killed at the same temperature/time ratio [31]. However, the acidity values at which this inactivation was achieved are very low and may cause syneresis of the product, as well as reduce consumer acceptance due to taste and appearance of the product. In addition, given that nutrients of protein nature and water-soluble vitamins are affected by heat treatment [30], it is of interest to consider other options that allow reducing heat exposure times, without the need to reach a high acidity content in the product.

Results obtained in this research could guide the answer to this problem. The enhanced effect of heat treatment in combination with medium and long chain monoacylglycerols does not require extreme reductions in the pH value of the product. It was shown that values close to neutrality (pH = 6.6) or slightly acidic (pH = 5.5) are sufficient to achieve a significant bactericidal effect. In this way, process parameters for the thermal treatments could be modified as a new hurdle technology. Redesigning thermal processes, applied to low-fat fermented products, would result in less severe treatments, ensuring the preservation of organoleptic characteristics and the decrease of nutritional deterioration. Consequently, heat exchangers with smaller heat transfer areas and shorter holding times would be required, with a considerable reduction in the costs associated to the thermal treatment stage of the product.

## Conclusions

The enhancing effect of heat treatment with medium- and long-chain monoacylglycerols could be exploited in more than one way for the preservation of processed foods. Their combined use could modify the control parameters of heat treatments, designing them as a new barrier preservation technology. In this way, the results obtained with lactic acid bacteria could guide the redesign of less severe thermal processes that would also extend the shelf life of low-fat fermented products, while helping to preserve their organoleptic characteristics and nutritional benefits, and reducing the costs associated with the heat treatment stage of the product.

## Data Availability

The datasets used and/or analysed during this study are also available from the corresponding author on reasonable request.
